# Ovarian Sertoli-Leydig Tumour in a Postmenopausal Female With Virilisation: Magnetic Resonance Imaging Findings

**DOI:** 10.7759/cureus.85965

**Published:** 2025-06-13

**Authors:** Yuya Yamamoto, Iichiro Osawa, Masao Takahashi, Kosei Hasegawa, Masanori Yasuda

**Affiliations:** 1 Radiology, Saitama Medical University Hospital, Moroyama, JPN; 2 Radiology, Saitama Medical University Hospital, Saitama, JPN; 3 Gynecologic Oncology, Saitama Medical University International Medical Center, Hidaka, JPN; 4 Pathology, Saitama Medical University International Medical Center, Hidaka, JPN

**Keywords:** magnetic resonance imaging, ovarian tumour, postmenopausal female, sertoli-leydig cell tumours, sex cord-stromal tumour, virilisation

## Abstract

Sertoli-Leydig cell tumours (SLCTs) of the ovary are uncommon sex cord-stromal tumours that may cause virilisation and menometrorrhagia. Herein, we describe the magnetic resonance imaging features of SLCT in a 75-year-old female who exhibited virilisation with elevated androgen and oestrogen levels. The tumour appeared as a well-defined multilobulated mass. Measuring 8 cm in diameter with flow voids and cavitation, and showed high signal intensity in the early and delayed phases. The uterine myometrium showed high signal intensity and a clear junctional zone on T2-weighted images, and the clitoris was enlarged. A solid ovarian mass demonstrating intense enhancement with flow voids associated with elevated oestrogen levels and virilisation could suggest SLCT.

## Introduction

Sertoli-Leydig cell tumour (SLCT) of the ovary is a rare type of sex cord-stromal tumour, accounting for less than 0.5% of all ovarian tumours [1]. This entity is histopathologically characterised by variable proportions of Sertoli and Leydig cells. Most SLCTs occur in young females with a mean age of 25 years, while less than 10% of patients with SLCT are over 50 years of age [1]. Clinical symptoms include those related to excess androgenic or oestrogenic hormones, and virilisation caused by androgen hyperproduction is the most common symptom. However, two-thirds of tumours do not exhibit any endocrine function [1]. Although poorly differentiated tumours may recur, SLCT has a good prognosis. Conservative surgery is acceptable for young patients wishing to preserve fertility, and postoperative adjuvant chemotherapy and long-term follow-up are recommended for those with high-risk factors [2]. Because of its rarity, only a few reports involving magnetic resonance imaging (MRI) have been published [3,4]. Here, we report a case of ovarian SLCT in a postmenopausal female with virilisation and discuss the MRI findings.

## Case presentation

A 75-year-old female (gravida 0, para 0) presented with a two-year history of alopecia on the scalp, facial hair growth, and voice deepening. Laboratory test results revealed elevated testosterone levels. These features are suggestive of virilisation and hyperandrogenism. The patient was referred to our hospital for further examination.

**Figure 1:**
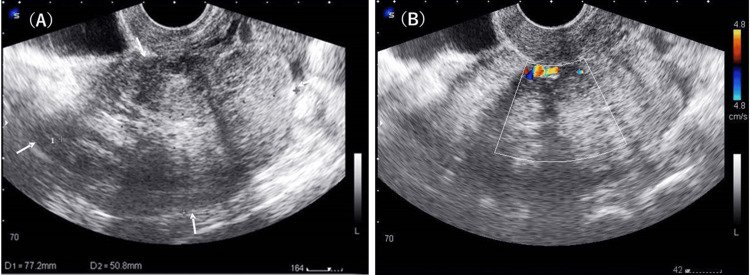
Transvaginal ultrasonography Transvaginal ultrasonography revealed a large, well-circumscribed solid mass (77 × 50 mm) in the left adnexa (A, arrows). This mass showed internal vascularity on colour Doppler imaging (B).

On physical examination, thinning of the scalp hair was associated with hair growth on the face and lower limbs. Pelvic examination revealed an enlarged clitoris and palpable mass in the pelvis. No evidence of vaginal bleeding was found. Laboratory testing revealed an elevated testosterone of 4.9 ng/mL (reference range: 0.11-0.47 ng/mL) and oestradiol level of 38 pg/mL (reference range: ≤ 21 pg/mL). All tumour markers, including cancer antigen (CA) 125, carbohydrate antigen (CA) 19-9, and carcinoembryonic antigen (CEA), were within the normal range. Transvaginal ultrasonography revealed a large, well-circumscribed mass (77 × 50 mm) in the left adnexa with internal vascularity on colour Doppler imaging (Figure 1A, 1B). There was no evidence of septations.

Non-contrast computed tomography (CT) demonstrated a large, well-circumscribed, lobulated mass in the left ovary. No calcification or tissue with fat density was observed within the mass. On contrast-enhanced CT, the mass showed heterogeneous enhancement and central fluid density. There was no evidence of ascites or metastasis (not shown).

**Figure 2:**
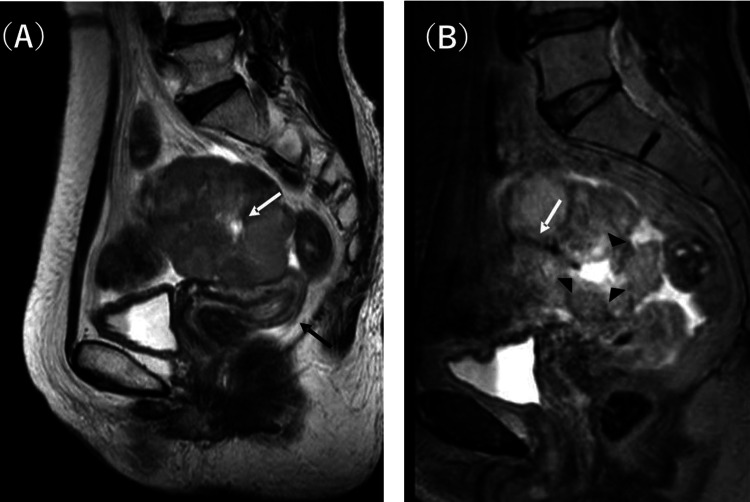
Sagittal T2-weighted imaging of the pelvis On non-contrast magnetic resonance images, a multilobulated, well-circumscribed, solid mass was noted in the left ovary, showing iso- to high signal intensity on T2-weighted imaging (T2WI) (A). The central part showed strong high signal intensity, suggestive of fluid collection (A, white arrow). The uterus was mildly enlarged for the menopausal age, with a distinct junctional zone and brighter myometrium (A, black arrow), suggestive of oestrogenic effects. Multiple flow voids in the mass were visualised more clearly on three-dimensional isotropic T2WI (B, white arrow), suggesting hypervascularity. The areas between the solid and central parts showed mildly high signal intensities (B, arrowheads).

**Figure 3:**
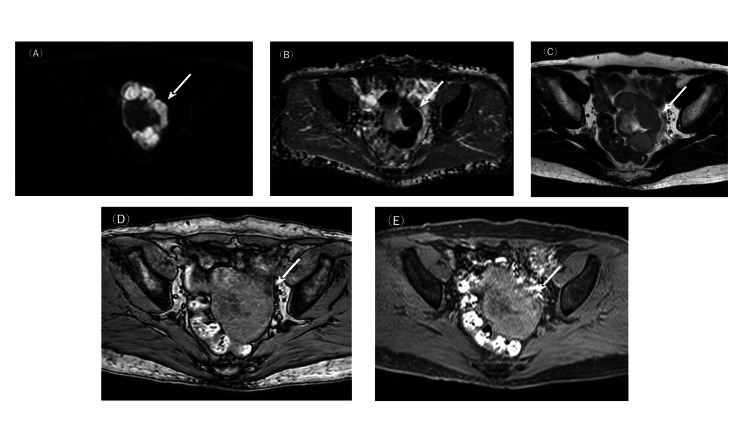
Axial magnetic resonance imaging of the pelvis The solid components showed high signal intensity on diffusion-weighted imaging (A), with low apparent diffusion coefficient values (B), corresponding to the mass on T2-weighted imaging (C). These components demonstrated low signal intensity on T1-weighted imaging (T1WI) (D) and pre-contrast fat-suppressed T1WI (E), with no evidence of fat or haemorrhage component.

This patient was scanned using a 3.0-T MRI scanner (Achieva dStream 3.0T, Philips Healthcare, Best, The Netherlands) with a phased-array multicoil in the supine position under free-breathing respiration. MRI revealed a multilobulated, well-circumscribed mass in the left ovary measuring 8 cm in diameter. The solid part showed iso- to high signal intensity compared to the outer myometrium on T2-weighted imaging (T2WI) (Figure 2A). The central part of the mass showed a strong high signal intensity (Figure 2A, 2B). The areas between these two regions showed mild high signal intensity (Figure 2B). The uterus was mildly enlarged (10 cm in the longest diameter) and the endometrium was mildly thickened (8 mm in thickness) considering the patient’s postmenopausal age, with a distinct junctional zone (not shown). In addition, the myometrium appeared relatively brighter on T2WI (Figure 2A). T1-weighted imaging (T1WI) demonstrated heterogeneous low signal intensity with multiple flow voids in the mass (not shown). Diffusion-weighted imaging (DWI) revealed high signal intensity peripherally (Figure 3A), with low apparent diffusion coefficient (ADC) values (Figure 3B). This restricted diffusion area was consistent with the solid components on T2WI (Figure 3C). T1WI and fat-suppressed T1WI showed no evidence of heamorrhage or fat component (Figure 3D, 3E).

**Figure 4:**
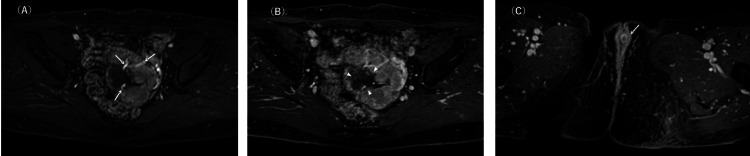
Dynamic contrast-enhanced magnetic resonance imaging of the pelvis On dynamic contrast-enhanced fat-suppressed T1-weighted imaging, the solid parts were intensely enhanced during the early phase (A), and the enhancement persisted in the delayed phase (B). The vasculature in the mass revealed prominent enhancement (A, arrows), corresponding to flow voids on non-contrast magnetic resonance imaging. The central part showed no enhancement and was more clearly visualised in the delayed phase (B, asterisk), indicating cavitation. The areas between these two parts were weakly enhanced (B, arrowheads), suggesting oedematous tissue. An enlarged clitoris was also noted (C, arrow).

On contrast-enhanced dynamic fat-suppressed T1WI, the solid parts were heterogeneously enhanced in the early phase (Figure 4A), and the enhancement persisted in the delayed phase (Figure 4B). No enhancement was observed in the central region. The areas between these two regions were mildly enhanced, together with a slightly high signal intensity on DWI. Clitoromegaly was also observed (Figure 4C). The right ovary appeared normal and no evidence of metastasis was observed. The patient underwent a total hysterectomy and bilateral salpingo-oophorectomy with omentectomy.

**Figure 5:**
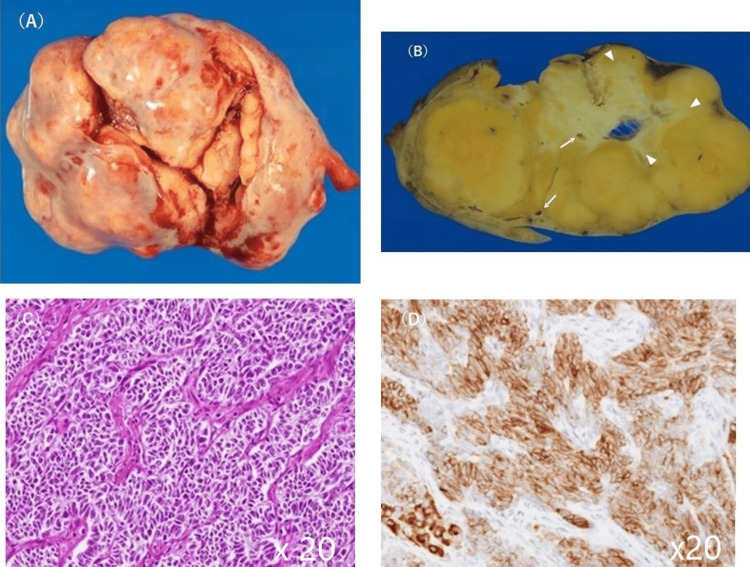
Ovarian pathology Gross photograph of the excised mass in the left ovary (A) showed a multilobulated mass. The cut surface of the excised mass in the left ovary was multilobulated and tan-yellow with central cavitation (B). The areas between the lobules and cavitation were white (B, arrowheads), indicating oedematous tissues. Increased vascularity was also observed within the mass (B, arrows). Haematoxylin and eosin staining showed numerous non-descript cells with a high nucleus-to-cytoplasm ratio and atypia, indicating Sertoli cells (C). Leydig cells were scattered among the Sertoli cells. Immunohistochemical staining revealed diffuse brown staining for positive result of inhibin-α (D), confirming the diagnosis of moderately differentiated Sertoli-Leydig cell tumours.

A gross photograph of the excised mass in the left ovary (Figure 5A) showed a multilobulated solid mass. The sectioned ovarian surface showed a tan-yellow multilobulated tumour with central cavitation (Figure 5B), which corresponded to a strong high signal intensity on T2WI. The areas between the lobules and cavitation were white, corresponding to mildly enhanced lesions on contrast-enhanced MRI and representing oedematous tissue. Intra-mass blood vessels were also visualised at the centre and periphery.

Microscopically, the lobules were dominated by non-descript cells with a high nucleus-to-cytoplasm ratio and atypia, indicating Sertoli cells (Figure 5C). Nests of Leydig cells with eosinophilic and vacuolated cytoplasm were interspersed in numerous Sertoli cells. Immunohistochemical analysis demonstrated that the tumour cells were positive for inhibin-α (Figure 5D). The constellation of histological features was consistent with moderately differentiated SLCT. The patient was discharged on the fifth hospital day uneventfully. Two years later, patient was followed up without treatment, serum testosterone and oestradiol levels returned to normal, and virilisation disappeared. There was no evidence of recurrence.

## Discussion

SLCT has sex cord and stromal elements that exhibit testicular differentiation. This tumour is typically unilateral, with only 1.5% involving both ovaries, and a mean diameter of 13.5 cm [1]. The most common symptom is virilisation caused by androgen hyperproduction, although hyperoestrogenism occasionally occurs [1]. Our patient also had high testosterone and oestradiol levels.

The virilising effects of the tumour were most probably caused by the accumulation of testosterone due to a deficiency in enzymes transforming testosterone to 17-ketosteroids or aromatisation to oestrogens [5]. In contrast, oestrogenic stimulation of the uterus may result from the peripheral aromatisation of testosterone to oestrogen, rather than primary tumour secretion [6].

The present case demonstrated associated uterine findings due to oestrogenic effects. In postmenopausal women, ovarian tumours with high oestradiol levels can show an enlarged uterus, thickened endometrium, and distinct junctional zone [7]. The combination of these three features in this case was highly suggestive of an oestrogenic effect. In contrast, an enlarged clitoris may be caused by androgenic effects. 

Cai et al. showed that the MRI features of SLCT can be roughly subdivided into three types: solid, solid and cystic, or cystic [8]. Solid types demonstrated iso-signal intensity on T2WI compared with the outer myometrium and intense high signal intensity on contrast enhancement. The MRI findings of solid-type tumours are similar to those in our case.

Ovarian tumours with elevated testosterone and oestrogen levels include SLCTs, steroid cell tumours, thecomas, granulosa cell tumours, sclerosing stromal tumours, and ovarian tumours with functioning stroma, including metastatic ovarian tumours [7]. Thecomas are the most common functional ovarian neoplasms. However, the higher signal intensity on T2WI and stronger enhancement make the diagnosis of thecoma unlikely. Adult granulosa cell tumours usually present with extensive haemorrhage or a white sponge-like appearance [9], which is atypical for SLCTs. Sclerosing stromal tumours may demonstrate pseudolobulation, which consists of low signal intensity nodules set against high signal intensity stroma on T2WI. Additionally, tumours can show early peripheral enhancement with centripetal progression on dynamic contrast-enhanced images [7]. The imaging features of metastatic ovarian tumours vary, including multilocular cystic masses and predominantly solid mass [10]. Metastatic tumours are often bilateral, which can help differentiate them from SLCTs. When metastatic tumours present with signs of excess androgen levels, the primary site is often apparent. Therefore, the detection of the primary site could be useful for diagnosing metastatic tumours.

The present case showed multiple flow voids within the tumour. Yamaoka et al. reported that yolk sac tumours showed a signal void on MRI and noted the presence of abundant vessels within the tumours [11]. In the present case, the tumour manifested as strongly enhanced masses and many dilated vessels on fat-suppressed T1WI, such as yolk sac tumours or sclerosing stromal tumours [11]. To the best of our knowledge, no prior MRI reports on SLCT with multiple voids have been published. Tumoural haemorrhage can occur in patients undergoing SLCT. Although multiple voids may mainly reflect blood flow in the vessels, some of these flow voids may be associated with haemorrhages within the tumour mass.

DWI characterises the restriction of Brownian movement of water molecules within tumours by using ADC values. Because ADC values are related to the translational movement of water molecules, increased tissue cellularity or cell density decreases the ADC value [12]. In general, malignant tumours have hypercellularity, enlarged nuclei, and angulation of the nuclear contour compared to benign lesions; therefore, ADC values assist in distinguishing between benign and malignant lesions [13,14]. Although multiple studies have examined the utility of DWI in the differential diagnosis of malignant from benign tumours, recent DWI studies have reported that they are not helpful in distinguishing between benign and malignant ovarian tumours [15-17]. Although most SLCTs behave in a benign nature, our case also showed high signal intensity on DWI and low ADC values on ADC map images, probably due to hypercellularity with the tumour and enlarged nuclei [15-17].

Testosterone-producing tumours showing signs of virilisation are rare, even in ovarian tumours, and are common in young people [1]; however, they can also occur in postmenopausal elderly females, and SLCT needs to be differentiated.

## Conclusions

In summary, we presented a case of SLCT in a postmenopausal woman with virilization. MRI findings, including moderate intense solid portions on T2WI, restricted diffusion, and hypervascularity presenting as flow voids and prominent enhancement, may provide useful information on diagnosis of SLCT. Although this tumour is rare, SLCT should be considered as a differential diagnosis in clinical situations where postmenopausal women with ovarian tumours show signs of virilization.
